# Population based cohort study of fetal deaths, and neonatal and perinatal mortality at term within a Somali diaspora

**DOI:** 10.1186/s12884-021-04163-z

**Published:** 2021-11-01

**Authors:** Stephen Contag, Rahel Nardos, Irina A. Buhimschi, Jennifer Almanza

**Affiliations:** 1 Department of Obstetrics, Gynecology and Women’s Health, University of Minnesota School of Medicine, Medical School MMC 395, 420 Delaware St SE, Minneapolis, MN 55455 USA; 2 Division of Female Pelvic Medicine and Reconstructive Surgery, Department of Obstetrics, Gynecology and Women’s Health, University of Minnesota School of Medicine, Global Women’s Health, Center for Global Health and Social Responsibility, Medical School MMC 395, 420 Delaware St SE, Minneapolis, MN 55455 USA; 3 Department of Obstetrics & Gynecology, University of Illinois at Chicago College of Medicine, Chicago, IL 60612 USA

**Keywords:** Fetal, Stillbirth, Neonatal, Death, Perinatal, Mortality, Somali, Term, Pregnancy

## Abstract

**Background:**

Somali women deliver at greater gestational age with limited information on the associated perinatal mortality. Our objective is to compare perinatal mortality among Somali women with the population rates.

**Methods:**

This is a retrospective cohort study from all births that occurred in Minnesota between 2011 and 2017. Information was obtained from certificates of birth, and neonatal and fetal death. Data was abstracted from 470,550 non-anomalous births ≥37 and ≤ 42 weeks of gestation. The study population included U.S. born White, U.S. born Black, women born in Somalia or self-identified as Somali, and women who identified as Hispanic regardless of place of birth (377,426). We excluded births < 37 weeks and > 42 weeks, > 1 fetus, age < 18 or > 45 years, or women of other ethnicities. The exposure was documented ethnicity or place of birth, and the outcomes were live birth, fetal death, neonatal death prior to 28 days, and perinatal mortality rates. These were calculated using binomial proportions with 95% confidence intervals and compared using odds ratios adjusted (aOR) for diabetes, hypertension and maternal body mass index.

**Results:**

The aOR [95%CI] for stillbirth rate in the Somali cohort was greater than for U.S. born White (2.05 [1.49–2.83]) and Hispanic women (1.90 [1.30–2.79]), but similar to U.S. born Black women (0.88 [0.57–1.34]). Neonatal death rates were greater than for U.S. born White (1.84 [1.36–2.48], U.S. born Black women (1.47 [1.04–2.06]) and Hispanic women (1.47 [1.05–2.06]). This did not change after analysis was restricted to those with spontaneous onset of labor. When analyzed by week, at 42 weeks Somali aOR for neonatal death was the same as for U.S. born White women, but compared against U.S. born Black and Hispanic women, was significantly lower.

**Conclusions:**

The later mean gestational age at delivery among women of Somali ethnicity is associated with greater overall risk for stillbirth and neonatal death rates at term, except compared against U.S. born Black women with whom stillbirth rates were not different. At 42 weeks, Somali neonatal mortality decreased and was comparable to that of the U.S. born White population and was lower than that of the other minorities.

**Supplementary Information:**

The online version contains supplementary material available at 10.1186/s12884-021-04163-z.

## Keypoints

**Question:** How does the greater median gestational age at delivery within the Somali diaspora impact perinatal mortality?

**Findings:** Perinatal mortality is increased mostly due to neonatal mortality, compared with US born White women, but comparable to that of other minorities. After 41 weeks, it is comparable to U.S. born White women.

**Meaning:** Perinatal mortality is similar among minorities due to worse neonatal outcomes rather than higher fetal death rates, and is greater when compared to U.S. born White women. After 41 weeks, Somali but no other minorities have outcomes similar those of U.S. born White women. This suggests that cultural and environmental resources may play a pivotal role in modifying neonatal outcomes.

## Introduction

Current epidemiological evidence shows that Somali women in the United States (U.S.) have lower rates of preterm birth. They carry their pregnancies to later gestational ages with a modal distribution of gestational age at delivery that is 1 week delayed compared with U.S. born white women [1] with higher rate of deliveries after 42 weeks [2]. This is also consistent with a cross-sectional population-based study of approximately 430,000 singleton births in Victoria, Australia comparing mothers born in East African countries relative to Australian-born women. As a group, East Africans had increased odds of small for gestational age births, very low birthweight and very preterm birth, yet decreased odds of preterm birth [3]. This was mostly due to decreased risk among Somali women, who had lower preterm birth rates compared with other East African and Australian women [3].

Conversely, studies show that immigrant women of African origin in Europe have the worst maternal and perinatal outcomes compared with a non-immigrant population [4]. Johnson et al. 2005 reported that maternal morbidity is increased among Somali immigrants in Washington State with cesarean delivery most often secondary to fetal distress and failed induction of labor. In their cohort, post term delivery rates and oligohydramnios were significantly increased compared with U.S. born White or Black women [2]. The mortality rate among babies born to immigrant women is not consistently higher in all countries, but in the U.S., it appears to be greater among immigrant Black women, including those from Somalia [3, 5, 6].

In a recent large epidemiologic study from Ohio that included almost 2,000,000 births from 2000 to 2015, rates of post term delivery after 42 weeks were increased fivefold among Somali women compared with all other ethnicities including African born women from West Africa [1]. This trend was maintained even after controlling for spontaneous or induced labor, smoking and parity [1]. It is not clear if this is associated with the observed increased perinatal morbidity and mortality reported for all immigrant Black women [7].

This situation is further complicated because Somali women will more often wait for spontaneous onset of labor (SOL) past 41 weeks before undergoing induction of labor [2].It is not clear whether this preference to delay delivery and waiting for SOL is associated with increased perinatal morbidity and mortality in the Somali diaspora resident in Minnesota.

Our hypothesis is that fetal, neonatal and perinatal mortality among Somali women is increased compared with U.S. born White, Black and Hispanic women in Minnesota secondary to an increased rate of deliveries after 40 weeks of gestation.

## Methods

This retrospective, large cohort study was performed with data obtained from linking live birth certificates with fetal death certificates for all births in the State of Minnesota between January 1, 2011 and December 31, 2017. Data was provided through an agreement between the Minnesota Department of Health and the University of Minnesota, and after obtaining Institutional Review Board (IRB) exemption from the University of Minnesota (IRB submission 00004909). The data agreement included all non-anomalous singleton births occurring at greater than 20 weeks between January 1, 2011 and December 31, 2017, to women who were residing in Minnesota during those years.

To address our hypothesis, the specific study aims were to compare the mean gestational age at delivery after SOL within the four largest ethnic and racial groups residing in the state based on categories used by the Centers for Disease Control and Prevention [8]. These are U.S. born white women, U.S. born Black women, Somali women and women of Hispanic ethnicity. We also compared the primary outcomes including fetal, neonatal and perinatal death rates at term from 2011 to 2017, between the four ethnic groups. This is a population-based cohort, and sample size was not calculated. Due to the small number of cases with missing data, we did not make any assumptions.

Inclusion criteria were singleton term pregnancies without any known genetic or congenital anomaly, between 37 and 42 weeks of gestation dated by best obstetrical estimate. We included all pregnancies of women who had either SOL or induction of labor. Exclusion criteria were women whose age was less than 18 or greater than 45 at time of delivery as these groups have been shown to have an associated increase in perinatal morbidity and mortality [9–11] and women who did not identify as belonging to one of the four ethnicities included in our study cohort. We also excluded women who delivered before 37 weeks or after 42 weeks of gestation. Delivery < 37 weeks is considered a preterm delivery and management of pregnancy > 42 weeks is not within our standard of care. This allowed us to analyze pregnancies delivered within the period defined as term [12]. We also excluded pregnancies with more than one fetus.

Descriptive characteristics for the groups were compared using chi-square or analysis of variance as indicated by either continuous or categorical data. The birth rates after SOL per week were determined using binomial proportions with 95% confidence intervals between 37 and 42 weeks for all 4 ethnic-racial groups. We calculated the weekly fetal death rates using total births (stillbirths and livebirths) as the denominator and cumulative fetal death rates (risk of fetal death among all ongoing pregnancies. Neonatal death rates were the proportion of infant deaths prior to 28 days of life among all live births and were calculated per week of gestation. The perinatal mortality rates per 10,000 births were calculated from the combined stillbirth and neonatal death rates using binomial proportions with 95% confidence intervals [13]. The risk for fetal and neonatal death regardless of whether labor was spontaneous or induced was calculated for all groups. A secondary analysis that only included women who delivered after SOL was performed to calculate risk for neonatal mortality but not for fetal death, as most are delivered after induction of labor and are not intrapartum events [14]. The methodology to classify women having SOL from birth certificates has been previously validated and published [15]. The odds ratio and adjusted odds ratio (aOR) for the primary outcomes was calculated between the various groups using the Somali population as a reference group and adjusting for clinically relevant factors available in the birth or fetal death certificates: body mass index, pregestational diabetes, pregestational hypertension, gestational diabetes and gestational hypertension. BMI was not consistently reported in all groups, but we did not make any assumption regarding BMI. Sensitivity analysis showed that excluding BMI did not change the results. We did not include access to state supplemented nutritional support and maternal level of education, as these are not reliably reported in the fetal or neonatal death certificates. A secondary analysis including nutritional support and maternal education (surrogates for socioeconomic status) was included in the supplemental tables. Birthweight was used to adjust odds ratios for neonatal death but not for fetal death as this information is not reliably reported among stillborn fetuses and is not readily available unless a livebirth has occurred. We analyzed the data using SAS/STAT® software 9.4 (Cary, NC) using a logit model, and applying a dichotomous outcome variable of fetal or neonatal death. The log odds of the outcome was modeled as a linear combination of the predictor variables included. All figures were created with GraphPad Prizm (GraphPad Prism version 8.0.0 for Windows, GraphPad Software, San Diego, California U.S.A, www.graphpad.com). This manuscript adhered to “Strengthening the Reporting of Observational Studies” (STROBE) guidelines for reporting cohort studies [16].

## Results

**Fig. 1:**
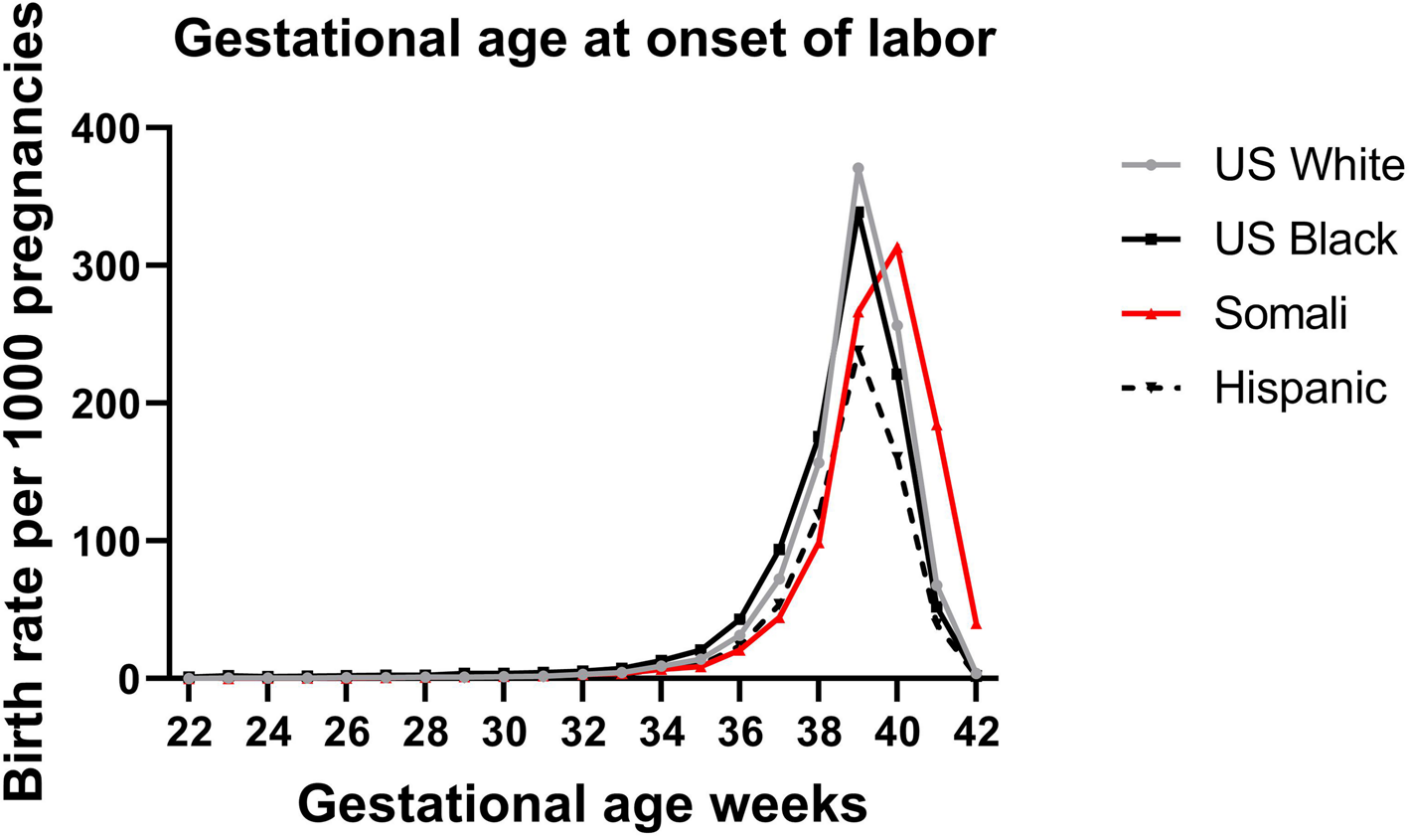
Birth rates distribution by gestational age after spontaneous onset of labor in Minnesota from 2011 to 2017 for U. S .born White, U.S. born Black, Somali and Hispanic women

**Fig. 2:**
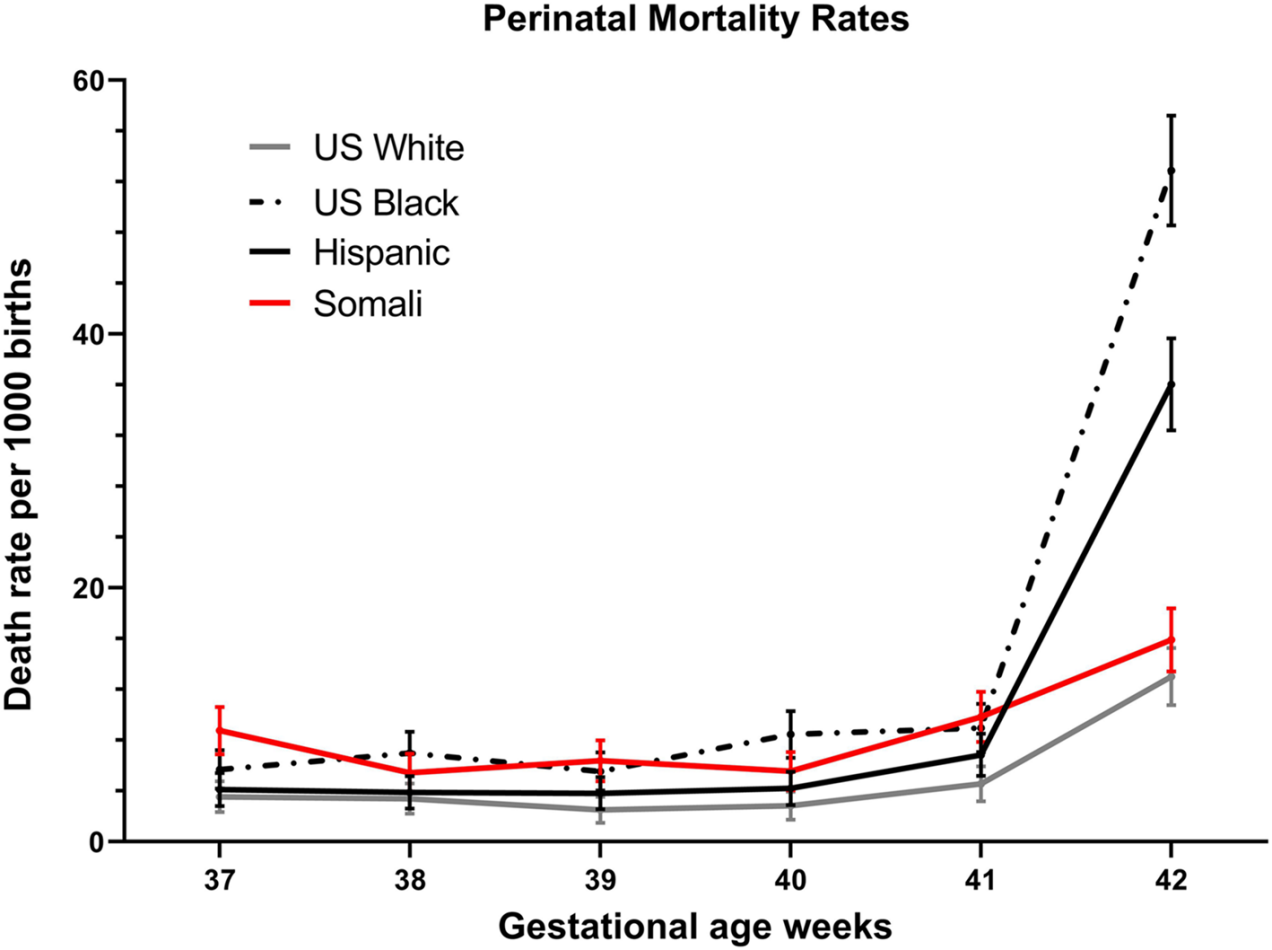
Perinatal mortality in Minnesota from 2011 to 2017: binomial rates with 95% confidence intervals for U. S .born White, U.S. born Black, Somali and Hispanic women

Records for 470,550 births of non-anomalous infants born in the State of Minnesota were provided for analysis. We excluded 16,479 births with more than 1 fetus, 33,383 that occurred prior to 37 or after 42 weeks, 13,526 for maternal age < 18 or > 45 years, and 29,744 women who did not identify as belonging to one of the ethnicities in the study cohort. Information for 377,426 total births and for 226,823 births after SOL were available for analysis. The mode for gestational age at delivery was 40 weeks for Somali women compared with 39 weeks for the rest of the population (Fig. 1). Perinatal mortality was higher compared with the U.S. born White population. However, compared against Hispanic and U.S. born Black women, perinatal mortality was higher between 37 and 40 weeks but lower after 41 weeks (Fig. 2).

We analyzed the births per year and observed a significant trend for decreasing birthrates among U.S. born White women while rates among U.S. born Black, Somali and Hispanic women were rising, with the second highest proportion observed among Hispanic women (supplemental Table 1).

When comparing the descriptive characteristics among ethnicities, Somali women tended to have a lower level of education and higher parity. Together with Hispanic women, Somali women had a higher frequency of gestational diabetes; however, they also had the lowest incidence of hypertensive disease of pregnancy (Table 1). Regarding delivery characteristics, Somali and Hispanic women had the highest rates of SOL. Although the highest frequency of vaginal delivery was observed among Hispanic women, both Somali and Hispanic women had the lowest primary cesarean delivery rates, with Somali women having the highest repeat cesarean delivery rates. Birthweight among Somali newborns was lower than that observed for U.S. born White women but higher than that observed for U.S. born Black and Hispanic women (Table 2).

**Table 1:** Maternal descriptive characteristics of study population: singleton term deliveries in Minnesota 2011–2017

	Ethnicity/Birthplace n (%)				
Maternal variables	U.S. White	U.S. Black	Somali	Hispanic	Total
**Age (years)** ^**a**^					
18–25	63,060 (21.1)	10,402 (51.9)	2333 (17.3)	18,599 (41.1)	94,394 (25.0)
26–35	201,024 (67.3)	8334 (41.6)	9194 (68.1)	22,370 (49.5)	240,922 (63.8)
36–45	34,581 (11.6)	1290 (6.4)	1976 (14.6)	4263 (9.4)	42,110 (11.2)
Total	298,665	20,026	13,503	45,232	377,426
Frequency Missing = 0					
**BMI Category (kg/m2)** ^**a**^					
Underweight	5784 (2.1)	420 (2.3)	443 (3.54)	846 (2.0)	7493 (2.1)
Normal	132,312 (47.0)	5718 (31.2)	4353 (34.8)	14,724 (35.5)	157,107 (44.4)
Overweight	75,093 (26.7)	4816 (26.3)	4339 (34.7)	12,978 (31.3)	97,226 (27.5)
Obese	68,142 (24.2)	7375 (40.2)	3387 (27.1)	12,918 (31.2)	91,822 (26.0)
Total	281,331	18,329	12,522	41,466	353,648
Frequency Missing = 23,778					
**Education** ^**a**^					
Less than high school	11,255 (3.8)	3484 (17.4)	7049 (52.2)	15,064 (33.3)	36,852 (9.8)
High school	40,922 (13.7)	6447 (32.2)	3232 (23.9)	12,692 (28.1)	63,293 (16.8)
College or Graduate	199,329 (66.7)	9440 (47.1)	3111 (23.0)	15,649 (34.6)	227,529 (60.3)
Post Graduate	47,140 (15.8)	651 (3.3)	107 (0.8)	1820 (4.0)	49,718 (13.2)
Unknown	19 (0.01)	4 (0.02)	4 (0.03)	7 (0.02)	34 (0.01)
Total	298,665	20,026	13,503	45,232	377,426
**Parity** ^**a**^					
Nulliparous	118,245 (39.7)	6849 (34.3)	2230 (16.6)	13,513 (30.0)	140,837 (37.4)
Parous	179,453 (60.3)	13,116 (65.7)	11,215 (83.4)	31,570 (70.0)	235,354 (62.6)
Total	297,698	19,965	13,445	45,083	376,191
Frequency Missing = 1235					
**Diabetes** ^**a**^					
No diabetes mellitus	281,101 (94.1)	18,730 (93.5)	12,189 (90.3)	40,476 (89.5)	352,496 (93.4)
Pregestational diabetes	1624 (0.5)	201 (1.0)	142 (1.1)	578 (1.3)	2545 (0.7)
Gestational diabetes	15,938 (5.3)	1095 (5.5)	1172 (8.7)	4177 (9.2)	22,382 (5.9)
Total	298,663	20,026	13,503	45,231	377,423
Frequency Missing = 3					
**Hypertension** ^**a**^					
No hypertension	280,109 (93.8)	18,204 (90.9)	13,047 (96.6)	42,937 (94.9)	354,297 (93.9)
Chronic hypertension	2890 (1.0)	517 (2.6)	56 (0.4)	417 (0.9)	3880 (1.0)
Gestational hypertension	15,662 (5.2)	1305 (6.5)	400 (3.0)	1878 (4.2)	19,245 (5.1)
Total	298,661	20,026	13,503	45,232	377,422
Frequency Missing = 4					

^a^: Chi square *P* < 0.01

^b^: ANOVA P < 0.01

**Table 2:** Delivery descriptive characteristics of study population: singleton term deliveries in Minnesota 2011–2017

	Ethnicity/Birthplace n (%)				
Neonatal variables	U.S. White	U.S. Black	Somali	Hispanic	Total
**Onset of Labor** ^**a**^					
Spontaneous	226,823 (76.2)	15,674 (78.5)	11,067 (82.4)	37,049 (82.1)	290,613 (77.2)
Induced	70,875 (23.8)	4293 (21.5)	2370 (17.6)	8063 (17.9)	85,601 (22.7)
Total	297,698	19,967	13,437	45,112	376,214
Frequency Missing = 1212					
**Gestational Age (weeks)** ^**a**^					
37	22,446 (7.5)	2067 (10.3)	625 (4.6)	3968 (8.8)	29,106 (7.7)
38	44,490 (14.9)	3670 (18.3)	1314 (9.7)	8156 (18.0)	57,630 (15.3)
39	116,605 (39.0)	7429 (37.1)	3568 (26.4)	17,027 (37.6)	144,629 (38.3)
40	81,528 (27.3)	4828 (24.1)	4169 (30.9)	11,629 (25.7)	102,154 (27.1)
41	31,790 (10.6)	1954 (9.8)	3044 (22.5)	4195 (9.3)	40,983 (10.9)
42	1806 (0.6)	78 (0.4)	783 (5.8)	257 (0.6)	2924 (0.8)
Total	298,665	20,026	13,503	45,232	377,426
Frequency Missing = 0					
**Sex**					
Male	152,354 (51.1)	10,078 (50.4)	6892 (51.2)	22,811 (50.5)	192,135 (51.0)
Female	145,972 (48.9)	9902 (49.6)	6569 (48.8)	22,351 (49.5)	184,794 (49.0)
Total	298,326	19,980	13,461	45,162	376,929
Frequency Missing = 497					
**Mode of Delivery** ^**a**^					
Vaginal delivery	206,320 (69.1)	13,963 (69.7)	9272 (68.7)	32,814 (72.6)	262,369 (69.5)
Vaginal birth after cesarean	13,525 (4.5)	601 (3.0)	436 (3.2)	1351 (3.0)	15,913 (4.2)
Primary cesarean delivery	73,775 (24.7)	4989 (24.9)	2873 (21.3)	9943 (22.0)	91,580 (24.3)
Repeat cesarean delivery	4840 (1.6)	449 (2.2)	905 (6.7)	1091 (2.4)	7285 (1.9)
Unknown	205 (0.1)	24 (0.1)	17 (0.1)	33 (0.1)	279 (0.1)
Total	298,665	20,026	13,503	45,232	377,426
**NICU admission** ^**a**^	17,324 (5.4)	2300 (10.3)	945 (6.6)	3191 (6.5)	
**Maternal Age (years)** ^**b**^					
Mean (SD)	30 (5)	26 (6)	30 (5)	27 (6)	
**Gestational Age (weeks)** ^**b**^					
Mean (SD)	39 (1)	39 (1)	40 (1)	39 (1)	
**Newborn Birthweight (grams)** ^**b**^					
Mean (SD)	3507 (461)	3257 (467)	3434 (460)	3399 (4667)	

^a^Chi square *P* < 0.01

^b^ ANOVA *P* < 0.01

*NICU* neonatal intensive care

Cumulative fetal death rates per week were comparable between all four ethnic groups up to 41 weeks, after which U.S. born Black and Hispanic fetal death rates were significantly higher compared with Somali and U.S. born White women (supplemental Figure1 ). Neonatal death rates followed a similar pattern. Somali neonatal death was comparable to all groups prior to 41 weeks, after which it remained lower compared with U.S. born Black and Hispanic women (supplemental Figure 1). NICU admission was also similar between all groups except for U.S. born Black women who had significantly higher NICU admission rates compared with the rest of the population (Table 2).

Cumulative fetal death rates among ongoing pregnancies were lower than neonatal death rates at less than 39 weeks; these risks converged at 39 weeks for U.S. born White, U.S. born Black and Hispanic women. This convergence was not observed until 40 weeks for Somali women. To demonstrate this effect, we have included a figure depicting the cumulative risk for stillbirth against the weekly neonatal death rate (supplemental Figure 2). After 40 weeks, risk of fetal death among Somali women became higher than the neonatal mortality rate, but still below the rates observed for women of Hispanic or U.S. born Black ethnicity.

The aOR for fetal death and neonatal death at term among Somali newborns was higher compared with U.S. born White (aOR, 2.05 [95%CI: 1.49–2.83], aOR 1.84 [95%CI: 1.36–2.48]) and Hispanic women (aOR, 1.90 [95%CI: 1.30–2.79] and 1.47 [95%CI: 1.05–2.06] respectively) (Tables 3 and 4 report unadjusted and aOR by week and for the entire cohort). When analysis was limited to neonatal deaths among women after SOL, the risk for neonatal death was still greater among Somali women (aOR, 1.79 [95%CI: 1.26–2.54]) compared with U.S. born White women, but not increased compared with Hispanic women (aOR, 1.42 [95%CI: 0.96–2.11]) (supplemental Table 2 also lists actual rates per week of gestation by ethnicity). There was no significant difference in risk for fetal death or neonatal death for all deliveries or after SOL compared with U.S. born Black women (aOR, 1.14 [95%CI: 0.75–1.74], 1.15 [95%CI: 0.80–1.66], and 1.05 [95%CI: 0.69–1.60] respectively). The aOR for fetal death among Somali women was not consistently different per week of gestation compared with the other three ethnic groups. The aOR for neonatal death among Somali women was higher compared with the U.S. born White births up to 40 weeks, after which there was no difference. Compared with U.S. born Black and Hispanic births, there was no difference until after 41 weeks, when the rates among Somali births were significantly lower.

We performed a secondary analysis that included maternal level of education and inclusion in the Special Supplemental Nutrition Program for Women, Infants, and Children (WIC) that provides federal grants to states for supplemental foods, health care referrals, and nutrition education for low-income pregnant, breastfeeding, and non-breastfeeding postpartum women, and to infants and children up to age five who are found to be at nutritional risk [17]. After adjusting for these surrogates of maternal socioeconomic status, we observed that the reported differences in stillbirth rates between Somali and U.S. born White women were no longer significant. This was also observed for neonatal death risk with no significant differences after adjusting for maternal education and participation in the WIC program (Supplemental Table 3).

**Table 3:** Odds ratios for fetal death in Minnesota from 2011 to 2017 for U.S. born White, U.S. born Black, Somali and Hispanic women

	Somali: U.S. White		Somali: U.S. Black		Somali: Hispanic	
Week	OR^**a**^ (95%CI)	aOR^**b**^ (95%CI)	OR (95%CI)	aOR (95%CI)	OR (95%CI)	aOR (95%CI)
**37**	2.95 (1.52–5.71)	2.17 (1.12–4.21)	1.39 (0.59–3.28)	1.08 (0.46–2.57)	1.81 (0.84–3.89)	1.72 (0.80–3.71)
**38**	0.43 (0.11–1.74)	0.32 (0.08–1.28)	0.41 (0.09–1.98)	0.33 (0.07–1.63)	0.24 (0.06–0.99)	0.22 (0.05–0.91)
**39**	2.41 (1.25–4.65)	1.87 (0.97–3.61)	0.80 (0.36–1.76)	0.66 (0.30–1.47)	2.94 (1.23–7.08)	2.67 (1.12–6.48)
**40**	2.40 (1.19–4.86)	1.85 (0.91–3.76)	0.89 (0.36–2.25)	0.72 (0.28–1.86)	1.87 (0.76–4.61)	1.79 (0.73–4.42)
**41**	3.17 (1.48–6.79)	2.19 (1.01–4.77)	2.51 (0.54–11.63)	1.71 (0.36–8.14)	3.62 (0.98–13.39)	3.00 (0.80–11.27)
**42**	0.77 (0.15–3.80)	0.66 (0.13–3.27)	0.21 (0.02–2.29)	0.21 (0.02–2.39)	0.34 (0.05–2.44)	0.32 (0.05–2.33)
**Total**	2.71 (1.97–3.74)	2.05 ((1.49–2.83)	1.40 (0.92–2.13)	0.88 (0.57–1.34)	2.01 (1.37–2.94)	1.90 (1.30–2.79)

^a^ (OR) Odds ratio

^b^ (aOR) Adjusted odds ratio by maternal BMI, pregestational diabetes, pregestational hypertension, gestational diabetes, gestational hypertension and birthweight

**Table 4:** Odds ratios for neonatal deaths < 28 days in Minnesota from 2011 to 2017 for U.S. born White, U.S. born Black, Somali and Hispanic women

	Somali: U.S. White		Somali: U.S. Black		Somali: Hispanic	
Week	OR^**a**^ (95%CI)	aOR^**b**^ (95%CI)	OR (95%CI)	aOR (95%CI)	OR (95%CI)	aOR (95%CI)
**37**	1.06 (0.73–1.52)	1.90 (1.29–2.82)	0.67 (0.35–1.30)	1.95 (0.95–4.0)	1.37 (0.98–1.92)	2.09 (1.13–3.86)
**38**	1.26 (0.90–1.76)	1.42 (1.01–1.99)	0.57 (0.35–0.94)	1.08 (0.64–1.83)	1.26 (0.77–2.08)	1.82 (1.10–3.02)
**39**	2.49 (1.80–3.45)	2.08 (1.50–2.89)	1.10 (0.68–1.81)	1.60 (0.97–2.66)	1.52 (0.99–2.31)	1.57 (1.03–2.40)
**40**	3.47 (2.42–4.97)	2.40 (1.65–3.51)	0.83 (0.50–1.39)	0.94 (0.55–1.60)	1.99 (1.19–3.34)	1.63 (0.96–2.74)
**41**	2.59 (1.71–3.91)	1.37 (0.88–2.14)	1.19 0.51–2.80)	1.01 (0.42–2.39)	1.08 (0.60–1.97)	0.73 (0.38–1.37)
**42**	1.04 (0.45–2.44)	0.54 (0.21–1.41)	0.14 (0.03–0.48)	0.10 (0.02–0.44)	0.18 (0.07–0.47)	0.08 (0.03–0.22)
**Total**	2.12 (1.57–2.86)	1.84 (1.36–2.48)	0.83 (0.58–1.18)	1.47 (1.04–2.06)	1.37 (0.98–1.92)	1.47 (1.05–2.06)

^a^ (OR) Odds ratio

^b^ (aOR) Adjusted odds ratio by maternal BMI, pregestational diabetes, pregestational hypertension, gestational diabetes, gestational hypertension and birthweight

## Discussion

This large population-based study demonstrated that the overall aOR for fetal death at term and for neonatal death prior to 28 days among Somali women was greater compared with U.S. born White and Hispanic women. Although the risk for fetal death was comparable to that of U.S. born Black women, the neonatal death rates were higher among Somali women compared with the other ethnic groups. When we analyzed risks according to the week of gestation, the higher risk for fetal and neonatal death rates among Somali women were no longer evident compared with U.S. born Black women. Based on these findings it would appear that greater mean gestational at delivery is associated with an increased risk for perinatal mortality among Somali women. The observed increased perinatal mortality observed among Somali women, was lower than that of the other two minority groups after 41 weeks.

In order to help us understand these differences we performed an adjusted analysis incorporating two surrogates for socioeconomic status: the reported level of maternal education and participation in the WIC program. After these adjustments fetal mortality among Somali women was not significantly different compared with U.S. born White and Black women, and slightly greater (1 to 3%) compared with Hispanic women. Neonatal mortality risk was not significantly different than any of the three other ethnic groups. These relationships did not change when we limited our analysis to women who delivered after SOL or after adjusting for surrogates of socioeconomic status. These findings suggest that although the increased perinatal mortality among Somali women may be a function of gestational age it also appears to be associated with socioeconomic status and possibly to disparities in access to health care. Interestingly adjusting for these surrogate variables did not change the decreased neonatal mortality at 42 weeks compared with other minorities.

Consistent with previous reports, mean gestational age at delivery after SOL among Somali women was delayed by 1 week compared with all other ethnicities. The increased post term birth rate reported by the Ohio study shows that the frequency of late term and post term births decreased after comparing Somali women born in Somalia to those born outside of Somalia [1]. Although early work suggested that this could be due to acculturation and willingness to follow recommendations when the health care provider recommended delivery, this has not been substantiated in subsequent studies [2].

It has been demonstrated that active management of pregnancies complicated with either maternal or neonatal comorbidities benefit from recommendations for early term or delivery by 39 weeks [18, 19]. Elective delivery by 39 weeks for low risk healthy women also results in lower rates of cesarean delivery, preeclampsia and neonatal morbidity [20, 21]. Our findings show that among Somali women, a longer duration of gestation is associated with increased perinatal mortality compared with U.S. born White and Hispanic women, but it was not increased compared with U.S. born Black women, who had similar fetal death rates and lower neonatal death rates. After 41 weeks, perinatal mortality increased for all four ethnicities, but was much greater for U.S. born Black and Hispanic women compared with Somali and US born White women.

Prior publications regarding pregnancies in the Somali population have been reported from the states of Washington, Ohio and Minnesota [1, 2, 22]. Research performed in Washington State and Ohio discusses increased rates of late term and post-term births among Somali women, with contradictory data regarding neonatal outcomes and fetal deaths [1, 2, 23]. Higher rates of adverse outcomes have been documented among Somali women having post term births [1, 2]. The Washington State study reported greater risk for adverse obstetrical and neonatal outcomes compared with both Black and White newborns [2]. Similar results were not observed from Ohio [1], where pregnancies were less often affected by hypertension yet more often complicated by diabetes, which is consistent with observations from Washington State and prior research done in Minnesota [2, 22].

As in this analysis, adverse perinatal outcomes in these previous studies were increased among Somali women, compared with U.S. born White women but not with U.S. born Black women or African born Black women. An important distinction between the two studies is that the earlier Washington study based gestational age on last menstrual period while the Ohio study based gestational age on the best obstetrical estimate [1, 2]. This difference may affect gestational age by as much as 2 to 3 weeks [24].

We did not specifically assess the impact of country of birth or time living in the U.S. among Somali women. Among African and Hispanic immigrant populations in the U.S., it has shown that immigrant women relative to U.S.-born women have a lower rate of preterm birth, lower birth weight and longer pregnancies [7, 25], after controlling for the effect of the community, culture, and environment, this effect was attenuated over time and is consistent with the results we obtained in secondary analysis [7]. The Ohio cohort study also noted that the observed lower rates of prematurity as well as the higher rate of post term birth decreased over time when comparing Somali women born in the U.S. with those born in Somalia [1]. Although we did not specifically address preterm birth rates in our study, in Fig. 1 it is evident that late preterm birth rates are lower among Somali women.

Our data does not include all outcomes reported in the previous studies; it does focus the discussion on fetal and neonatal deaths in the Somali population and which is consistent with what we know about increasing perinatal mortality with greater gestational age past 39 weeks. Although most studies have compared Somali neonatal deaths to the U.S. born White population, the evidence is not clear whether the neonatal death rates observed, which are are also greater than those of U.S. born Black and Hispanic women are different after adjusting for minority or immigrant status or disparities in access to health care [26–28].

Despite having the largest Somali diaspora in the country [1] there has been limited information on late term and post term birth rates within this group in Minnesota. Although trends showing the effect of acculturation have reported greater maternal weight gain and preterm births rates, this is limited to early or first generation Somali women [22]. Our current analysis has allowed us to evaluate a longer-term effect of acculturation, environment and access to care, now that there is a generation of Somali women who have been born in the U.S. and are having children of their own.

The data show that despite acculturation, mean gestational age at delivery is delayed by 1 week within the Somali population compared with the rest of the population. Previous work in Minnesota compared Somali women delivering between 1993 and 1999 with those who delivered between 2000 and 2006 [22]. The authors measured factors hypothesized to reflect acculturation and their relationship to the higher preterm birth rates expected over time among immigrant Somali women. The factors evaluated and thought to reflect acculturation and affect birth outcomes among Somali women such as age at immigration, years lived in the U.S. and English language proficiency did not account for the observed increase in the preterm birth rate. However increased incidence of maternal obesity and gestational diabetes did, suggesting that diet and obesity may play a larger role in the observed increasing rate of preterm birth in this population, while prenatal care utilization prevented preterm birth [22]. The association of pregnancy outcome with gestational diabetes (GDM) is consistent with a large meta-analysis of over 120,000 women that evaluated the risk for GDM among migrant women compared with the non-migrant population, and found that they had higher rates of GDM than non-immigrant women [29].

Determining whether initiation of birth was spontaneous or by induction of labor, cesarean without labor or membrane rupture is critical in surveillance and etiological research on preterm birth [15]. The same holds true for post term birth and risks for adverse perinatal outcome [19]. Whether factors influencing preterm birth rates can affect post term birth rates remains to be determined. If factors such as diet and the development of GDM can affect the overall duration of pregnancy and timing of SOL, Somali women would provide an excellent reference to evaluate how factors such as dietary changes over time could change perinatal outcomes by shortening duration of pregnancy. It remains to be seen what factors are influencing neonatal mortality in the minority populations included, but as suggested in our secondary analysis, it is likely that cultural and socioeconomic factors play a large role especially regarding access to care.

In conclusion, Somali women on average delivered later than the rest of the population. This delay is associated with fetal and neonatal death rates that are increased compared with the rest of the population. The only fetal death rates that were similar were those of the U.S. born Black population which historically are higher than for the rest of the population. These differences were not apparent after correcting for surrogates of socioeconomic status. At 42 weeks, neonatal mortality decreased compared with other minorities and was comparable to that of the U.S. born White population and this did not change after adjusting for socioeconomic status. This information is consistent with previous work demonstrating that minority and immigrant populations have been and continue to be at risk for adverse perinatal outcomes most likely due to disparities in access to care. These findings provide a framework within which to evaluate biological, cultural, and factors related to access to care that can affect duration of pregnancy and neonatal outcomes.

## Supplementary Information

**Additional file 1.**

## Data Availability

The data that support the findings of this study are available from the Minnesota Department of Health through submission of a Data Use Agreement, but restrictions apply to the availability of these data, which were used under license for the current study, and so are not publicly available. Data are however available from the authors upon reasonable request and with permission of the Minnesota Department of Health. The Minnesota Department of Health will not release individual level data to protect individuals who could be identified from areas with small numbers of live births, fetal or neonatal deaths. The summary data from which the calculations are derived are available in Table 2 of the supplemental data.

## Abbreviations

aOR: Adjusted odds ratio
U.S.: United States of America
CI: Confidence interval
SOL: Spontaneous onset of labor
GDM: Gestational diabetes mellitus

## References

[1] Oliver EA, Klebanoff M, Yossef-Salameh L (2018). Preterm birth and gestational length in four race-nativity groups, Including Somali Americans. Obstet Gynecol.

[2] Johnson EB, Reed SD, Hitti J, Batra M (2005). Increased risk of adverse pregnancy outcome among Somali immigrants in Washington state. Am J Obstet Gynecol.

[3] Belihu FB, Davey MA, Small R (2016). Perinatal health outcomes of east African immigrant populations in Victoria, Australia: a population based study. BMC Pregnancy Childbirth.

[4] Malin M, Gissler M (2009). Maternal care and birth outcomes among ethnic minority women in Finland. BMC Public Health.

[5] Calderon-Margalit R, Sherman D, Manor O, Kurzweil Y (2015). Adverse Perinatal Outcomes among Immigrant Women from Ethiopia in Israel. Birth (Berkeley, Calif).

[6] Gissler M, Alexander S, MacFarlane A (2009). Fetal deaths and infant deaths among migrants in industrialized countries. Acta Obstet Gynecol Scand.

[7] Elsayed A, Amutah-Onukagha NN, Navin L, Gittens-Williams L, Janevic T. Impact of immigration and duration of residence in US on length of gestation among black women in Newark, New Jersey. J Immigr Minor Health. 2018. 10.1007/s10903-018-0813-7 PMC9994288 30171430

[8] Centers for DIsease Control and Prevention (CDC). Pregnancy Mortality Surveillance System. 2020; https://www.cdc.gov/reproductivehealth/maternal-mortality/pregnancy-mortality-surveillance-system.htm. Accessed 18 June 2020.

[9] Leader J, Bajwa A, Lanes A (2018). The effect of very advanced maternal age on maternal and neonatal outcomes: a systematic review. J Obstet Gynaecol Can.

[10] Pinheiro RL, Areia AL, Mota Pinto A, Donato H (2019). Advanced maternal age: adverse outcomes of pregnancy, a Meta-analysis. Acta Medica Portuguesa.

[11] Ganchimeg T, Ota E, Morisaki N (2014). Pregnancy and childbirth outcomes among adolescent mothers: a World Health Organization multicountry study. BJOG.

[12] American College of Obstetrics and Gynecology. Practice bulletin no. 130: prediction and prevention of preterm birth. Obstet Gynecol 2012;120(4):964–973, DOI: 10.1097/AOG.0b013e3182723b1b. 10.1097/AOG.0b013e3182723b1b 22996126

[13] Smith GCS (2001). Life-table analysis of the risk of perinatal death at term and post term in singleton pregnancies. Am J Obstetr Gynecol.

[14] Getahun D, Ananth CV, Kinzler WL (2007). Risk factors for antepartum and intrapartum fetal death: a population-based study. Am J Obstet Gynecol.

[15] Klebanoff MA, Yossef-Salameh L, Latimer C (2016). Development and validation of an algorithm to determine spontaneous versus provider-initiated preterm birth in US vital records. Paediatr Perinat Epidemiol.

[16] von Elm E, Altman DG, Egger M, Pocock SJ, Gøtzsche PC, Vandenbroucke JP. The Strengthening the Reporting of Observational Studies in Epidemiology (STROBE) Statement: guidelines for reporting observational studies. Int J Surgery (London, England). 2014;12(12):1495–1499. 10.1016/j.ijsu.2014.07.013 25046131

[17] Agriculture USDo. Special supplemental nutrition program for women, infants, and children (WIC). 2021; https://www.fns.usda.gov/wic. 12814069

[18] Nicholson JM, Parry S, Caughey AB, Rosen S, Keen A, Macones GA (2008). The impact of the active management of risk in pregnancy at term on birth outcomes: a randomized clinical trial. Am J Obstetr Gynecol.

[19] American College of Obstetrics and Gynecology (2014). Practice bulletin no. 146: management of late-term and postterm pregnancies. Obstet Gynecol.

[20] Society of Maternal Fetal Medicine. SMFM Statement on Elective Induction of Labor in Low-Risk Nulliparous Women at Term: The ARRIVE Trial. American journal of obstetrics and gynecology. 2018. 10.1016/j.ajog.2018.08.009 30098985

[21] Grobman WA, Rice MM, Reddy UM (2018). Labor induction versus expectant Management in low-Risk Nulliparous Women. N Engl J Med.

[22] Flynn PM, Foster EM, Brost BC (2011). Indicators of acculturation related to Somali refugee women's birth outcomes in Minnesota. J Immigr Minor Health.

[23] Rassjo EB, Byrskog U, Samir R, Klingberg-Allvin M (2013). Somali women's use of maternity health services and the outcome of their pregnancies: a descriptive study comparing Somali immigrants with native-born Swedish women. Sexual Reprod Healthcare.

[24] American College of Obstetricians and Gynecologists (2017). Committee opinion no 700: methods for estimating the due date. Obstet Gynecol.

[25] Flores ME, Simonsen SE, Manuck TA, Dyer JM, Turok DK (2012). The "Latina epidemiologic paradox": contrasting patterns of adverse birth outcomes in U.S.-born and foreign-born Latinas. Women's Health Issues.

[26] Grobman WA, Bailit JL, Rice MM (2015). Racial and ethnic disparities in maternal morbidity and obstetric care. Obstet Gynecol.

[27] Urquia ML, Qiao Y, Ray JG, Liu C, Hjern A (2015). Birth outcomes of foreign-born, native-born, and mixed couples in Sweden. Paediatr Perinat Epidemiol.

[28] Small R, Gagnon A, Gissler M (2008). Somali women and their pregnancy outcomes postmigration: data from six receiving countries. BJOG.

[29] Gagnon AJ, McDermott S, Rigol-Chachamovich J, Bandyopadhyay M, Stray-Pedersen B, Stewart D (2011). International migration and gestational diabetes mellitus: a systematic review of the literature and meta-analysis. Paediatr Perinat Epidemiol.

