# Supplementary material for: Population based cohort study of fetal deaths, and neonatal and perinatal mortality at term within a Somali diaspora
Source: BMC Pregnancy Childbirth. 2021 Nov 1;21:740. doi: 10.1186/s12884-021-04163-z (PMC8559350; doi:10.1186/s12884-021-04163-z)

**Title:** **Population based cohort study of fetal deaths, and neonatal and perinatal mortality at term within a Somali diaspora**

**Stephen Contag, M.D., Rahel Nardos, MD, MCR, Irina A. Buhimschi, M.D. MMS., Jennifer Almanza, DNP, APRN, CNM**

**Supplemental Tables and Figures:**

**Supplemental Table 1:** Total births and births after spontaneous onset of labor per year by ethnicity. Panel A includes all deliveries. Panel B births after the spontaneous onset of labor.

| **Table 1A: Total births per year by ethnicity ^a^** | | | | | |
| --- | --- | --- | --- | --- | --- |
|  | Ethnicity n (%) | | | |  |
| Year | US White | US Black | Somali | Hispanic | Total |
| 2011 | 43580 (81.0) | 2619 (4.9) | 1488 (2.8) | 6144 (11.4) | 53801 |
| 2012 | 42902 (80.0) | 2722 (5.1) | 1652 (3.1) | 6349 (11.8) | 53625 |
| 2013 | 43237 (79.8) | 2898 (5.4) | 1745 (3.2) | 6338 (11.7) | 54218 |
| 2014 | 43031 (79.2) | 2934 (5.4) | 1963 (3.6) | 6433 (11.8) | 54361 |
| 2015 | 42733 (78.5) | 2918 (5.4) | 2124 (3.9) | 6676 (12.3) | 54451 |
| 2016 | 42298 (78.1) | 2929 (5.4) | 2250 (4.2) | 6658 (12.3) | 54135 |
| 2017 | 40884 (77.4) | 3006 (5.7) | 2281 (4.3) | 6664 (12.6) | 52835 |
| Total | 298665 (79.1) | 20026 (5.3) | 13503 (3.6) | 45232 (12.0) | 377426 |
|  |  |  |  |  |  |
| a: Cochrane Armitage test for trend P-value <0.01 . Chi square for entire cohort P-value <0.01 | | | | | |

|  | | |  |  |  |
| --- | --- | --- | --- | --- | --- |
|  |  | | | |  |
|  |  |  |  |  |  |
|  |  |  |  |  |  |
|  |  |  |  |  |  |
|  |  |  |  |  |  |
|  |  |  |  |  |  |
|  |  |  |  |  |  |
|  |  |  |  |  |  |
|  |  |  |  |  |  |
|  |  |  |  |  |  |
|  |  |  |  |  |  |
|  | | | | |  |
|  |  |  |  |  |  |

| **Table 1B: Births after spontaneous onset of labor per year by ethnicity ^a^** | | | | | |
| --- | --- | --- | --- | --- | --- |
|  | Ethnicity n (%) | | | |  |
| Year | US White | US Black | Somali | Hispanic | Total |
| 2011 | 33436 (79.9) | 2080 (5.0) | 1219 (2.9) | 5091 (12.2) | 41826 |
| 2012 | 32614 (78.7) | 2202 (5.3) | 1356 (3.3) | 5275 (12.7) | 41447 |
| 2013 | 33202 (78.7) | 2284 (5.4) | 1430 (3.4) | 5260 (12.5) | 42176 |
| 2014 | 32911 (78.1) | 2271 (5.4) | 1626 (3.9) | 5330 (12.7) | 42138 |
| 2015 | 32441 (77.5) | 2253 (5.4) | 1719 (4.1) | 5468 (13.1) | 41881 |
| 2016 | 31815 (77.1) | 2254 (5.5) | 1863 (4.5) | 5326 (12.9) | 41258 |
| 2017 | 30404 (76.2) | 2330 (5.8) | 1854 (4.7) | 5299 (13.3) | 39887 |
| Total | 226823 (78.0) | 15674 (5.4) | 11067 (3.8) | 37049 (12.7) | 290613 |
|  |  |  |  |  |  |
| a: Cochrane Armitage test for trend P-value <0.01 . Chi square for entire cohort P-value <0.01 | | | | | |

Supplemental Table 2: Odds ratios for stillbirth and neonatal deaths in Minnesota from 2011-2017 for U.S. born White, U.S. born Black, Somali and Hispanic women. Neonatal deaths were analyzed for all births and after spontaneous onset of labor. Panel A: stillbirths. Panel B: neonatal deaths after all births. Panel C: neonatal deaths after spontaneous onset of labor.

A:

| **Table for fetal death with odds ratio comparing women of Somali ethnicity to U.S. white, U.S. Black and Hispanic ethnicities** | | | | | | | |
| --- | --- | --- | --- | --- | --- | --- | --- |
|  | **Events by Ethnicity (stillbirths: total births)** | | | | **Adjusted OR (95% CI)^a^** | | |
| **Week** | **U.S. White** | **U.S. Black** | **Somali** | **Hispanic** | **Somali : U.S. White** | **Somali : U.S. Black** | **Somali : Hispanic** |
| **37** | 75:22464 | 11:2126 | 10:619 | 19:4055 | 2.17 (1.12 - 4.21) | 1.08 (0.46 - 2.57) | 1.72 (0.80 - 3.71) |
| **38** | 100:44601 | 7:3793 | 2:1315 | 28:8360 | 0.32 (0.08 - 1.28) | 0.33 (0.07 - 1.63) | 0.22 (0.05 - 0.91) |
| **39** | 83:117010 | 16:7629 | 10:3564 | 10:17472 | 1.87 (0.97 - 3.61) | 0.66 (0.30 - 1.47) | 2.67 (1.12 - 6.48) |
| **40** | 54:81973 | 9:5025 | 9:4177 | 10:12066 | 1.85 (0.91 - 3.76) | 0.72 (0.28 - 1.86) | 1.79 (0.73 - 4.42) |
| **41** | 25:32048 | 2:2064 | 9:3059 | 3:4373 | 2.19 (1.01 - 4.77) | 1.71 (0.36 - 8.14) | 3.00 (0.80 - 11.27) |
| **42** | 6:1802 | 1:79 | 2:785 | 2:264 | 0.66 (0.13 - 3.27) | 0.21 (0.02 - 2.39) | 0.32 (0.05 - 2.33) |
| a: Adjusting for maternal BMI, pregestational diabetes, pregestational hypertension, gestational diabetes, and gestational hypertension. | | | | | | | |

B:

| **Table for neonatal death <28 days all births with odds ratio comparing women of Somali ethnicity to U.S. white, U.S. Black and Hispanic ethnicities** | | | | | | | |
| --- | --- | --- | --- | --- | --- | --- | --- |
|  | **Events by Ethnicity (neonatal death: livebirths)** | | | | **Adjusted OR (95% CI)^a^** | | |
| **Week** | **U.S. White** | **U.S. Black** | **Somali** | **Hispanic** | **Somali : U.S. White** | **Somali : U.S. Black** | **Somali : Hispanic** |
| **37** | 74:22464 | 11:2126 | 5:619 | 15:4055 | 1.90 (1.29 - 2.82) | 1.95 (0.95 - 4.0) | 2.09 (1.13 - 3.86) |
| **38** | 123:44601 | 23:3793 | 6:1315 | 23:8360 | 1.42 (1.01 - 1.99) | 1.08 (0.64 - 1.83) | 1.82 (1.10 - 3.02) |
| **39** | 163:117010 | 24:7629 | 16:3564 | 40:17472 | 2.08 (1.50 - 2.89) | 1.60 (0.97 - 2.66) | 1.57 (1.03 - 2.40) |
| **40** | 82:81973 | 21:5025 | 9:4177 | 21:12066 | 2.40 (1.65 - 3.51) | 0.94 (0.55 - 1.60) | 1.63 (0.96 - 2.74) |
| **41** | 43:32048 | 6:2064 | 8:3059 | 14:4373 | 1.37 (0.88 - 2.14) | 1.01 (0.42 - 2.39) | 0.73 (0.38 - 1.37) |
| **42** | 6:1802 | 2:79 | 3:785 | 5:264 | 0.54 (0.21 - 1.41) | 0.10 (0.02 - 0.44) | 0.08 (0.03 - 0.22) |
| a: Adjusting for maternal BMI, pregestational diabetes, pregestational hypertension, gestational diabetes, gestational hypertension and birthweight. | | | | | | | |

C:

| **Table for neonatal death <28 days after spontaneous onset of labor with odds ratio comparing women of Somali ethnicity to U.S. white, U.S. Black and Hispanic ethnicities** | | | | | | | |
| --- | --- | --- | --- | --- | --- | --- | --- |
|  | **Events by Ethnicity (neonatal death : livebirths)** | | | | **Adjusted OR (95% CI)^a^** | | |
| **Week** | **U.S. White** | **U.S. Black** | **Somali** | **Hispanic** | **Somali : U.S. White** | **Somali : U.S. Black** | **Somali : Hispanic** |
| **37** | 59:17731 | 9:1719 | 3:517 | 12:3346 | 1.66 (1.06 - 2.59) | 1.45 (0.65 - 3.23) | 1.75 (0.87 - 3.53) |
| **38** | 98:38433 | 15:3221 | 4:1153 | 20:7404 | 1.36 (0.92 - 2.00) | 1.14 (0.61 - 2.16) | 1.60 (0.92 - 2.79) |
| **39** | 113:90975 | 19:6217 | 15:3107 | 32:14817 | 2.14 (1.46 - 3.15) | 1.48 (0.83 - 2.62) | 1.52 (0.94 - 2.46) |
| **40** | 58:62915 | 18:4049 | 7:3666 | 17:10000 | 2.35 (1.51 - 3.66) | 0.79 (0.45 - 1.41) | 1.62 (0.90 - 2.91) |
| **41** | 21:16628 | 3:956 | 6:2155 | 7:2449 | 1.60 (0.89 - 2.87) | 0.90 (0.27 - 2.96) | 0.74 (0.32 - 1.70) |
| **42** | 3:935 | 0:31 | 0:470 | 2:125 | 0.57 (0.13 - 2.46) | n/a | 0.11 (0.03 - 0.48) |
| a: Adjusting for maternal BMI, pregestational diabetes, pregestational hypertension, gestational diabetes, gestational hypertension and birthweight. | | | | | | | |

Supplemental Table 3: Adjusted odds ratios for fetal death and neonatal deaths <28 days in Minnesota from 2011-2017 for U.S. born White, U.S. born Black, Somali and Hispanic women including WIC^d^ usage and maternal level of education.

|  | **Adjusted OR Fetal death (95% CI)^a,c^** | | | **Adjusted OR Neonatal death (95% CI)^b,c^** | | |
| --- | --- | --- | --- | --- | --- | --- |
| **Week** | **Somali : U.S. White** | **Somali : U.S. Black** | **Somali : Hispanic** | **Somali : U.S. White** | **Somali : U.S. Black** | **Somali : Hispanic** |
| **37** | 1.56 (0.65 – 3.76) | 0.98 (0.36 – 2.71) | 1.60 (0.69 – 3.71) | 1.30 (0.78 – 2.17) | 1.46 (0.68 – 3.11) | 1.65 (0.87 – 3.12) |
| **38** | 0.25 (0.06 – 1.13) | 1.84 (0.23 – 15.02) | 0.24 (0.06 – 1.00 | 1.07 (0.69 – 1.68) | 0.88 (0.50 – 1.54) | 1.61 (0.94 – 2.76) |
| **39** | 1.12 (0.47 – 2.66) | 0.77 (0.28 – 2.14) | 3.50 (1.16 – 10.54) | 1.35 (0.88 – 2.06) | 1.14 (0.66 – 1.98) | 1.37 (0.87 – 2.16) |
| **40** | 2.47 (0.97 – 6.26) | 0.73 (0.24 – 2.18) | 2.19 (0.81 – 5.93) | 1.57 (0.96 – 2.58) | 0.78 (0.44 – 1.37) | 1.43 (0.83 – 2.45) |
| **41** | 1.39 (0.42 – 4.58) | 3.45 (0.34 – 35.05) | 2.88 (0.56 – 14.94) | 0.93 (0.52 – 1.66) | 1.04 (0.40 – 2.72) | 0.73 (0.37 – 1.47) |
| **42** | 0.40 (0.04 – 3.92) | 0.16 (0.01 – 3.55) | 0.27 (0.04 – 1.99) | 0.45 (0.14 – 1.45) | 0.08 (0.02 – 0.37) | 0.09 (0.03 – 0.25) |
| **Total** | 1.50 (0.97 – 2.31) | 1.29 (0.75 – 2.20) | 1.02 (1.01 – 1.03) | 1.18 (0.82 – 1.69) | 0.91 (0.60 – 1.36) | 1.28 (0.89 – 1.85) |
| a:(OR) Odds ratio. b: (aOR) Adjusted odds ratio by maternal BMI, WIC use, maternal education, pregestational diabetes, pregestational hypertension, gestational diabetes, gestational hypertension and birthweight. c: (CI) Confidence interval. d: WIC is the Women, Infants and Children food supplementation program from the U.S. Department of Agriculture. | | | | | | |

Supplemental Figure 1: Fetal and neonatal death rates in Minnesota from 2011-2017: Upper left panel: neonatal death <28 days rates with 95% confidence intervals. Lower left panel: fetal death rates with 95% confidence intervals. Upper right panel: corresponding 2^nd^ degree polynomial regressions derived from the neonatal death rates. Lower right panel: corresponding 2^nd^ degree polynomial regressions derived from the fetal death rates.


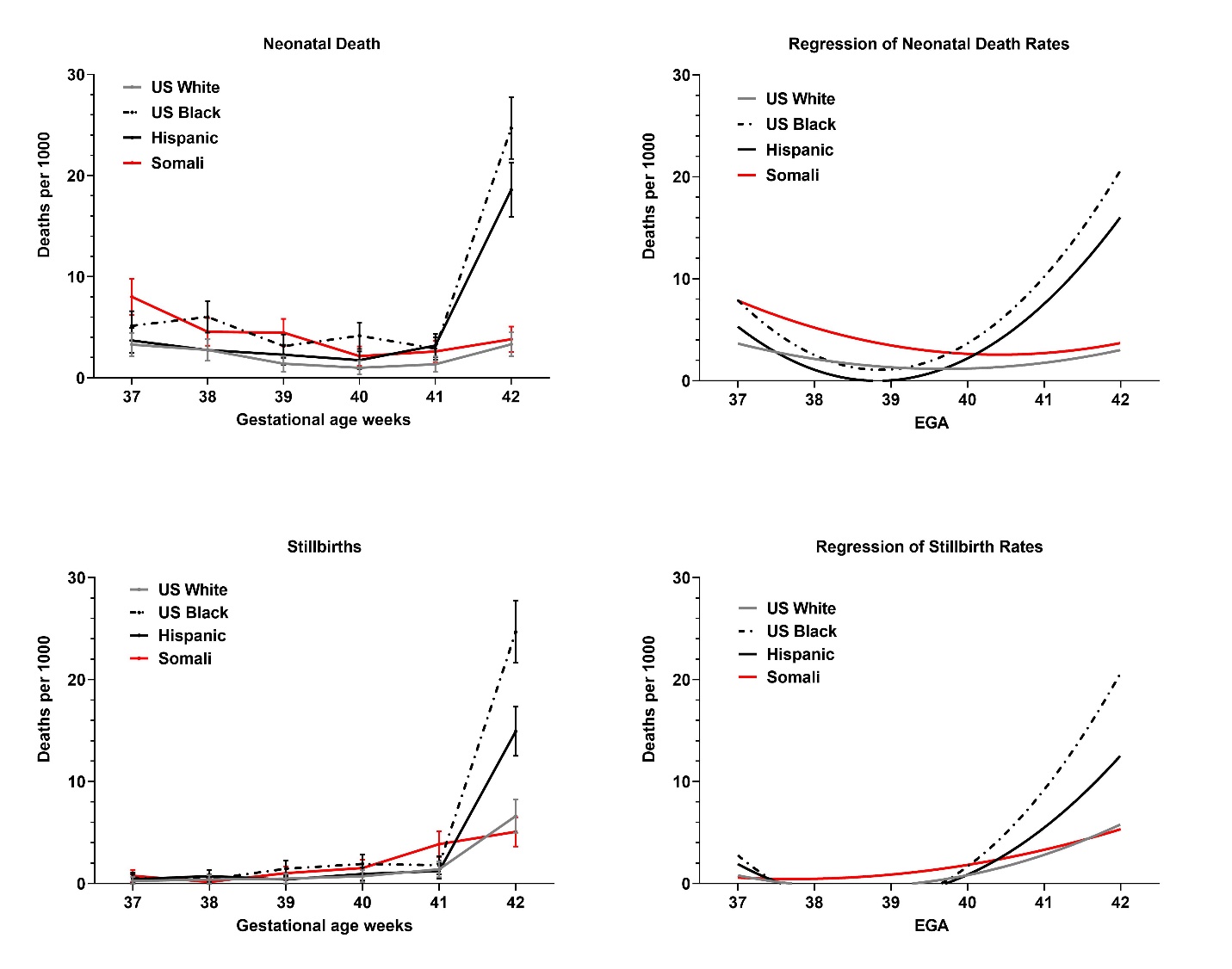


Supplemental Figure 2: Comparison of cumulative risk for stillbirth and neonatal death rates per 1,000 births at each week of gestation for all Minnesota births for years from 2011-2017 by ethnic group category. Neonatal deaths are the proportion of infant deaths within 28 days of birth among all live births; fetal deaths are the proportion of stillbirths among total births.


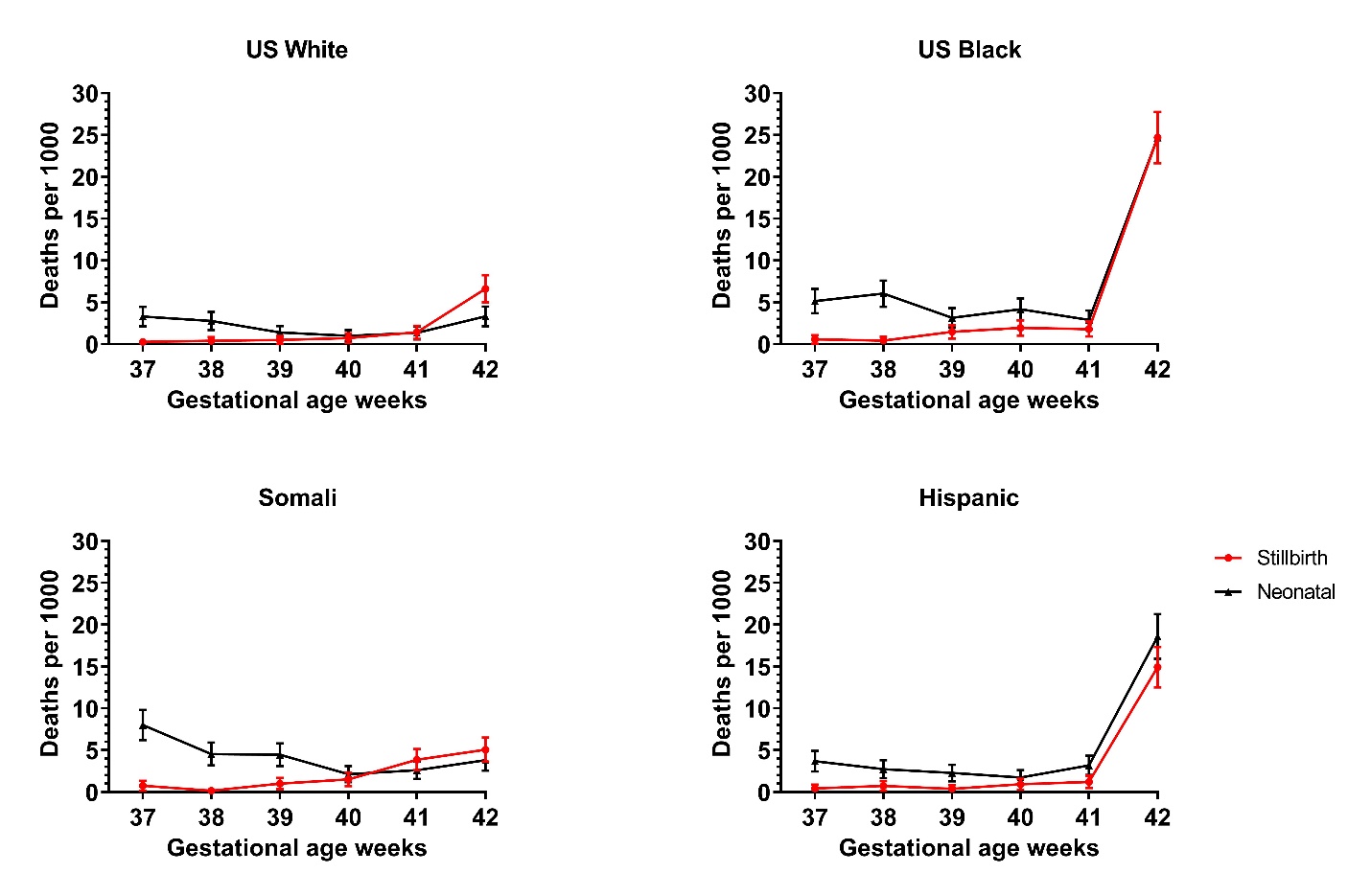

Supplement: Supplementary file 1 — Additional file 1. [file 12884_2021_4163_MOESM1_ESM.docx]
